# Proteomic evidence of dietary sources in ancient dental calculus

**DOI:** 10.1098/rspb.2018.0977

**Published:** 2018-07-18

**Authors:** Jessica Hendy, Christina Warinner, Abigail Bouwman, Matthew J. Collins, Sarah Fiddyment, Roman Fischer, Richard Hagan, Courtney A. Hofman, Malin Holst, Eros Chaves, Lauren Klaus, Greger Larson, Meaghan Mackie, Krista McGrath, Amy Z. Mundorff, Anita Radini, Huiyun Rao, Christian Trachsel, Irina M. Velsko, Camilla F. Speller

**Affiliations:** 1 Department of Archaeology, Max Planck Institute for the Science of Human History, Jena, Germany; 2 Department of Archaeogenetics, Max Planck Institute for the Science of Human History, Jena, Germany; 3 BioArCh, Department of Archaeology, University of York, York, UK; 4 Laboratories of Molecular Anthropology and Microbiome Research, Department of Anthropology, University of Oklahoma, Norman, USA; 5 Institute for Evolutionary Medicine, ETH-Zürich, University of Zürich, Zürich, Switzerland; 6 Functional Genomics Center, ETH-Zürich, University of Zürich, Zürich, Switzerland; 7 Department of Periodontology, College of Dentistry, University of Oklahoma Health Sciences Center, Oklahoma, OK, USA; 8 EvoGenomics, Natural History Museum of Denmark, University of Copenhagen, Copenhagen, Denmark; 9 Novo Nordisk Foundation Center for Protein Research, Faculty of Health and Medical Sciences, University of Copenhagen, Copenhagen, Denmark; 10 Discovery Proteomics Facility, Target Discovery Institute, University of Oxford, Oxford, UK; 11 The Palaeogenomics and Bio-Archaeology Research Network, Research Laboratory for Archaeology and the History of Art, University of Oxford, Oxford, UK; 12 York Osteoarchaeology Ltd, Bishop Wilton, York, UK; 13 Pinellas Dental Specialties, Largo, FL 33776, USA; 14 Department of Anthropology, College of Arts and Sciences, University of Tennessee, Knoxville, TN, USA; 15 Key Laboratory of Vertebrate Evolution and Human Origins of Chinese Academy of Sciences, Institute of Vertebrate Paleontology and Paleoanthropology, Chinese Academy of Sciences, Beijing, People's Republic of China; 16 Department of Anthropology, University of British Columbia, Vancouver, BC, Canada

**Keywords:** proteomics, dental calculus, dietary reconstruction, mass spectrometry

## Abstract

Archaeological dental calculus has emerged as a rich source of ancient biomolecules, including proteins. Previous analyses of proteins extracted from ancient dental calculus revealed the presence of the dietary milk protein β-lactoglobulin, providing direct evidence of dairy consumption in the archaeological record. However, the potential for calculus to preserve other food-related proteins has not yet been systematically explored. Here we analyse shotgun metaproteomic data from 100 archaeological dental calculus samples ranging from the Iron Age to the post-medieval period (eighth century BC to nineteenth century AD) in England, as well as 14 dental calculus samples from contemporary dental patients and recently deceased individuals, to characterize the range and extent of dietary proteins preserved in dental calculus. In addition to milk proteins, we detect proteomic evidence of foodstuffs such as cereals and plant products, as well as the digestive enzyme salivary amylase. We discuss the importance of optimized protein extraction methods, data analysis approaches and authentication strategies in the identification of dietary proteins from archaeological dental calculus. This study demonstrates that proteomic approaches can robustly identify foodstuffs in the archaeological record that are typically under-represented due to their poor macroscopic preservation.

## 1. Introduction

Archaeological dental calculus is a rich source of ancient DNA and proteins, providing insights into past oral microbial communities [1,2] and ancient diets [3]. Dental plaque accumulates on the tooth surface during life and, in the presence of calcium and phosphate ions in saliva and gingival crevicular fluid, mineralizes to form dental calculus (tartar) [4,5]. In doing so, dental calculus entombs and preserves biomolecules associated with the oral microbiota [1,2,6], the host [7], and inhaled and/or ingested microdebris [8], including environmental or occupational debris [8,9] and food particles such as starches and phytoliths [10–14]. Specifically, traces of foodstuffs can be sourced directly from the human mouth, uniquely revealing precise evidence of particular foods consumed, as opposed to evidence of food preparation (e.g. from residues on ceramic vessels) or of bulk diet (e.g. stable isotope analysis). In addition, dental calculus harbours favourable conditions for biomolecular preservation, given that biomolecules become entrapped rapidly by mineralization *in situ* and are thus relatively protected from environmental alteration during the postmortem interval [15].

Many foodstuffs are under-represented in the archaeological record due to poor preservation of diagnostic tissues. While microscopic fragments of these foodstuffs may persist in dental calculus, as well as in soils, ceramics and other objects of material culture (such as grindstones), taxonomic identification can be challenging. Plant microfossils (e.g. phytoliths, starch granules, pollen) are frequently non-diagnostic or only identifiable at a high level of taxonomic rank, such as kingdom (e.g. monocot) or family (e.g. Poaceae), and secondary animal products (e.g. milk, eggs) may leave little or no visible archaeological traces. By contrast, proteins are robust and highly diagnostic molecules that can survive for thousands to millions of years in archaeological and paleontological contexts [16,17]. Moreover, proteins are often expressed in specific tissues, allowing the different parts of plants (e.g. seeds versus leaves) [18] and animals (e.g. muscle versus milk) [3] to be distinguished. If such diagnostic proteins are preserved, they may more precisely identify foodstuffs compared with other lines of archaeological evidence, such as faunal remains, ancient DNA and stable isotope analysis.

In terms of dietary reconstruction, the analysis of ancient proteins is revealing new insights into the identification of past foodstuffs and vessel contents. Examples include the identification of protein residues adhering to vessels [19–21], and the ingredients in preserved remains of sourdough bread [22] and fermented milk products [23]. These approaches have been particularly promising in contexts which favour biomolecular preservation, such as anaerobic, waterlogged [24], cold [18] and arid conditions [20,25]. The recent discovery of preserved milk proteins within ancient archaeological dental calculus [3] further extends dietary protein recovery beyond the analysis of extraordinary finds from unusually well-preserved contexts, to a substrate that routinely preserves in many skeletal assemblages. Although many dietary DNA sources have been reported in calculus [2,26], to date, only a single class of dietary proteins (i.e. milk) has been investigated.

**Figure 1.**
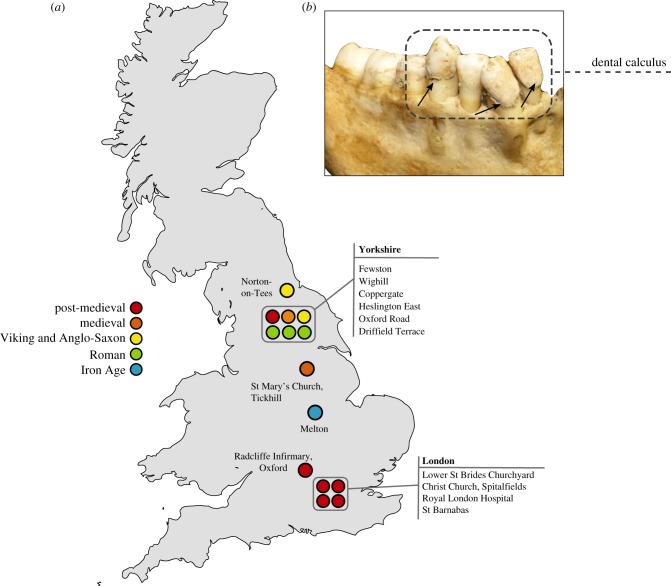
Map of archaeological dental calculus samples analysed in this study. (*a*) Map of Great Britain showing distribution of archaeological sites analysed in this study, colour-coded by time period. Specific details on the archaeological sites analysed in this study, including site codes and repository IDs, can be found in electronic supplementary material, table S2. Norton-on-Tees refers to two archaeological sites—East Mill and Bishopsmill School. (*b*) Example of dental calculus analysed in this study (Lower St Brides, SK1932). (Online version in colour.)

In order to explore this question further we reanalyse 38 previously published shotgun proteomics datasets from the Iron Age through to the Victorian period in England [3] (figure 1). We then apply a newly developed protein extraction method, gel-aided sample preparation (GASP) [27], to 62 dental calculus samples from eighteenth- and nineteenth-century England. Finally, we analyse proteins identified in 14 samples of modern dental calculus in order to explore the presence and preservation of dietary proteins in contemporary samples.

## 2. Material and methods

### (a) Summary

Detailed methodological information on protein extraction and identification can be found in the electronic supplementary material. Provenience information for the archaeological skeletons may be found in electronic supplementary material, table S2. A total of 100 archaeological samples of dental calculus were analysed, along with an additional 14 dental calculus deposits from living (*n* = 10) or recently deceased (*n* = 4) individuals. Of the total 114 samples analysed in this study 76 are newly generated datasets and 38 are a re-analysis of publicly available raw mass spectra data from Warinner *et al*. [3] (electronic supplementary material, table S1). The 38 previously published samples, as well as the 14 modern samples extracted in this study underwent a modified filter-aided sample preparation protocol (FASP) previously published in Warinner *et al*. [2,3]. For the 62 new archaeological samples from the post-medieval period, we applied a GASP based on Fischer & Kessler [27]. This protocol involves demineralizing the dental calculus matrix, followed by the copolymerization of solubilized proteins with acrylamide, enabling reduction, alkylation and enzymatic digestion; like the FASP method, GASP is designed to deal with high SDS abundance. For two of the modern samples, we applied both the FASP and the GASP extraction methods to compare the efficacy of both methods. Spectral data were converted to Mascot generic format (mgf) and LC–MS/MS ion database searching was performed on Mascot (Matrix Science, v. 2.4.01), against UniProt and the Human Oral Microbiome database (HOMD). Searches were performed against a decoyed database to generate protein false discovery rates, which were adjusted to less than 5%. Protein results were filtered to an ion score of greater than 25, and containing a minimum of two distinct peptides matching to different regions of the protein. Based on initial Mascot results we assigned protein identifications into the following classifications: contaminants, human, non-human animals and plants. We took a conservative approach and assigned any protein identified in our blank controls or injection blanks to the ‘contaminant' category. Initially, all non-human animal and plant proteins were considered as potential dietary proteins, and were further interrogated using BLAST (NCBI). Any non-human animal or plant peptides that also matched identically to human or microbial proteins were not considered as possible dietary proteins. Likewise, any non-human animal or plant peptides deriving from proteins identified within the ‘contaminant' dataset (electronic supplementary material, table S4) were also eliminated as potential dietary proteins.

## 3. Results and discussion

### (a) Identified proteins

**Figure 2.**
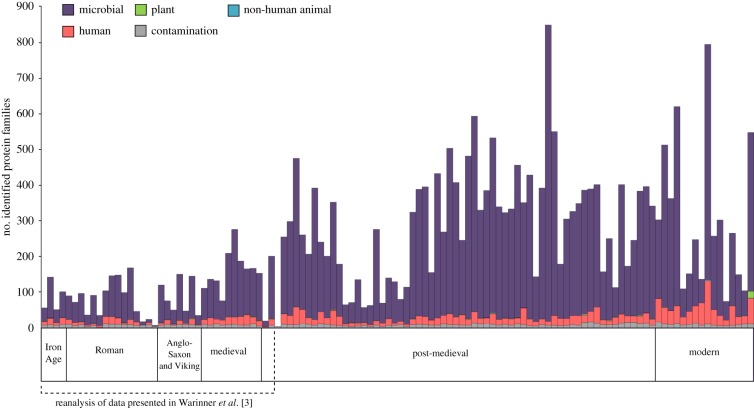
Number of identified proteins in modern and ancient dental calculus assigned to broad taxonomic categories of microbiota, the human host, non-human animals, plants and potential laboratory contaminants (prior to downstream confirmation of putative dietary proteins). Data include 76 new samples and re-analysis of 38 raw data files published in Warinner *et al.* [3]. (Online version in colour.)

**Figure 3.**
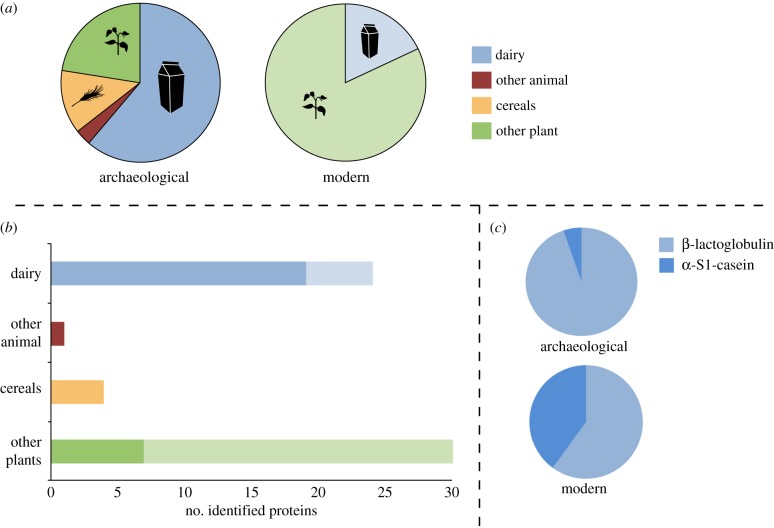
Dietary protein sources identified from samples of archaeological (*n* = 26) and modern (*n* = 4) dental calculus. (*a*) Proportion of identified dietary proteins assigned to plant and animal sources. (*b*) The total number of identified dietary proteins from dental calculus (darker hues signify archaeological samples; light hues signify modern samples). (*c*) The proportion of α-S1-casein (curd) and β-lactoglobulin (whey) milk proteins identified in archaeological and modern dental calculus samples. (Online version in colour.)

We identified a total of 59 putative dietary proteins in the entire assemblage, with 31 detected in archaeological samples, and 28 found in modern samples (electronic supplementary material, table S3). Overall, we found a low proportion of putative dietary proteins when compared with microbial and human components (figure 2), with 26 of 100 archaeological samples and 4 of 14 modern samples yielding at least one dietary protein. In archaeological samples, putative dietary proteins were dominated by dairy proteins (figure 3). The whey protein β-lactoglobulin was the most commonly identified dietary protein, present in 19 of the 100 archaeological samples. After excluding all collagens, keratins and egg-derived proteins as possible laboratory contaminants, only a single protein, haemoglobin deriving from the Pecora infraorder (i.e. ruminant) could be confidently assigned as a non-dairy animal protein. Plant proteins, including those from oats (*Avena sativa*), peas (*Pisum sativum*) and cruciferous vegetables (*Brassica* spp.), were also identified in the archaeological samples. In modern samples (*n* = 14), we identified a suite of plant proteins, including potato (*Solanum tuberosum*), soybean (*Glycine* sp.) and peanut (*Arachis hypogea*), as well as milk proteins. In one remarkable modern sample (Z100), 432 putative dietary peptides were identified, with 82% of these derived from six different peanut proteins, which may suggest that this dental patient consumed peanuts near to the time of periodic plaque mineralization or just prior to calculus collection. No dietary-derived proteins were detected in any laboratory extraction blanks (*n* = 9), which, in contrast to the dental calculus profiles, were dominated by trypsin, human skin proteins (e.g. collagens, keratins, dermcidin) as well as conserved microbial peptides, which could rarely be resolved to species.

### (b) Protein diagenesis and individual variation

We observe that the relative proportion of putative dietary proteins within dental calculus is low compared with microbial and human proteins (figure 2). Within the ancient and modern datasets, microbial and human proteins account for 38–98% (mean 83%) and 0–25% (mean 9.9%) of identified protein families, respectively. This finding is consistent with previous proteomic analyses of dental calculus [2] and with the fact that dental calculus is a calcified plaque biofilm. In contrast, non-human animals and plants represent 0–2% (mean 0.4%) of identified protein families within the archaeological samples and 0–3.5% (mean 0.3%) in the modern samples (figure 2; electronic supplementary material, table S3). In spite of their relatively low frequency, putative dietary proteins were identified in all time periods, spanning the Iron Age to the post-medieval period, suggesting they are not prone to selective loss from the system. On average, 35% of individuals from any given archaeological site produced dietary information, and only three sites yielded no putative dietary proteins (absent in Heslington East, Roman, *n* = 2; Tickhill, medieval, *n* = 4; Radcliffe Infirmary, post-medieval, *n* = 10). Even within the modern dataset, only 28% of individuals displayed dietary proteins, suggesting that the sporadic detection of dietary information is not linked exclusively to taphonomic factors, and that dietary proteins may not be observable in all individuals using current methods.

**Figure 4.**
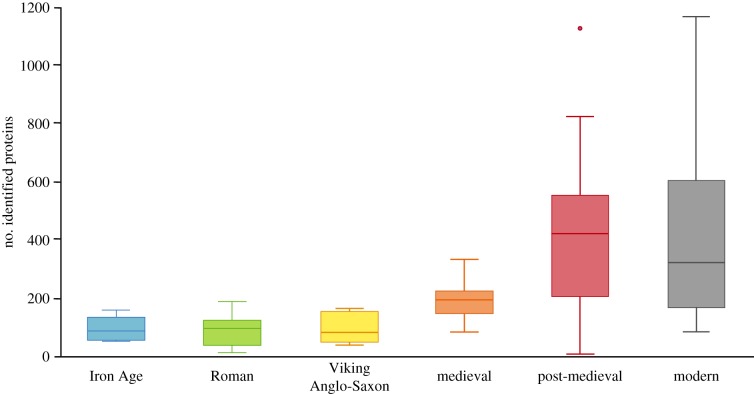
Average number of total identified proteins per sample by time period (box plots display 95% confidence interval). One post-medieval individual in particular, seen as the outlier, yielded a very high number of identified proteins (1123), comparable with the number of proteins identified from modern samples. Samples from the Iron Age, Roman, Viking and Anglo-Saxon, medieval and two post-medieval periods are a re-analysis of a previously published dataset [3]. (Online version in colour.)

Our temporal transect of dental calculus samples recovered from a consistent geographical area (England) provides insights into broad-scale trends in archaeological protein preservation (figure 4). There was a statistically significant difference in total protein identifications between time periods as determined by one-way ANOVA (*F*_5,110_ = 8.898, *p* < 0.0005); however, Games-Howell post hoc tests revealed that this was primarily due to significantly higher average number of protein identifications in the GASP-extracted post-medieval samples compared with all previous time periods (*p* < 0.0005). When only those samples extracted using the FASP protocol were compared, modern samples had significantly higher protein yields compared with samples from the Iron Age, Roman and Anglo-Saxon periods (*p* = 0.016, *p* = 0.012, *p* = 0.015, respectively), but not the medieval period (*p* = 0.147), suggesting a gradual decline in protein preservation over time and highlighting the fundamental importance of the sample preparation method. In spite of this taphonomic trend, the detection of dietary proteins remains relatively constant through time, with only a single modern individual exhibiting an ‘over-representation' of dietary proteins (specifically, an unusually high number of peanut proteins identified in sample Z100; electronic supplementary material, figure S4). Interestingly, we observe that milk proteins are consistently detected throughout all time periods and within 20% of ancient and modern individuals overall.

In addition to broad temporal trends in protein preservation, our analyses also displayed inter-site and intra-site differences in overall protein preservation. To assess inter-site differences in overall protein preservation we compared the total number of protein identifications in individuals from contemporaneous time periods which were extracted using the same methodology. Independent *t*-tests revealed no significant differences between the two Roman period sites of Oxford Road and Driffield Terrace (*t*_10_ = −0.384, *p* = 0.709), but significant differences between the two medieval sites of Wighill and Tickhill (*t*_8_ = 3.876, *p* = 0.005). Within the post-medieval period, no differences could be observed in the overall protein identifications among Fewston (North Yorkshire), St Brides Farringdon and Spitalfields (London); however, a one-way ANOVA revealed significant differences between Royal London Hospital and all other sites (*F*_3,47_ = 15.144, *p* = 0.001). The inconsistencies in overall protein yield between contemporaneous sites within a confined geographical area suggest that site-specific taphonomic factors and/or skeletal curation conditions also influence overall protein yields. We also detect high levels of individual variation in terms of total and dietary protein identifications, with no correlation between the quantity (mg) of dental calculus analysed per individual and the total number of identified proteins within that sample (electronic supplementary material, figure S1), an observation also noted by Mackie *et al*. [28]. High inter-individual variation in the total number of identified proteins was observed at all sites and across all time periods, including in modern samples, suggesting that the detection of putative dietary proteins using current methods is stochastic, and that the failure to identify dietary proteins for a given sample cannot be attributed to consumption habits or poor preservation alone. Little is currently known about how dietary proteins become trapped within dental calculus, and variation in this process may influence downstream protein recovery and identification success. Until we understand the degradation of these proteins, we cannot conclude that the absence of evidence is the evidence of absence.

The stochastic nature of dietary evidence in calculus is not an issue confined to proteins; similar site- or population-level variation has been reported for plant phytolith and starch microparticles [29,30], as well as dietary DNA in dental calculus [2,26]. In spite of the serendipitous nature of their detection, however, dietary proteins offer valuable information for identifying foodstuffs, such as dairy products, meats and certain edible plant tissues, that are otherwise not be detectable or distinguishable using microscopy or DNA.

### (c) Animal proteins

Milk proteins were the most frequently identified dietary proteins in archaeological dental calculus samples. Only ruminant milk proteins were detected, and peptide sequences enabled taxonomic assignment at various levels, including the infraorder Pecora (ruminants, *n* = 1), the family Bovidae (bovids, *n* = 9) and the subfamily Bovinae (cattle, bison, water buffalo, *n* = 9). Among archaeological samples, milk proteins were dominated by the whey protein β-lactoglobulin (BLG, as previously described in [3]), and only one post-medieval sample (FAO18) contained a non-BLG milk protein—caprine specific α-S1-casein, the most abundant protein in ruminant milk. In modern dental calculus samples, milk proteins were detected in 4 of 14 samples, including three identifications of β-lactoglobulin and two of α-S1-casein. No other milk proteins were identified.

It is unclear why only these two milk proteins have been detected in dental calculus, or why β-lactoglobulin, a protein found in far lower abundance in whole milk than α-S1-casein, is typically the only milk protein identified in archaeological samples. β-lactoglobulin is known to be a highly robust protein with enhanced resistance to enzymatic digestion [31], as well as microbial proteolysis [32,33], and heat and acid denaturation [34], which may contribute to its long-term survival in the archaeological record [3]. Further research is necessary to understand the mechanisms of protein, and especially milk protein, preservation within dental calculus; nevertheless, these results highlight the utility of the proteomic analysis of dental calculus for identifying the consumption of dairy foods.

Proteomic evidence for the consumption of non-dairy animal products was limited to one putative faunal blood protein: a haemoglobin protein of likely ruminant origin identified in a single post-medieval individual (FAO14). This underrepresentation of non-dairy derived animal proteins is strongly influenced by the fact that many of the most abundant proteins in muscle, skin, and bone (i.e. collagens, keratins, actin and myosin) are expressed by highly conserved genes with little or no sequence variation, and thus peptides deriving from these proteins may not be resolvable below the taxonomic level of class or order. When such dietary proteins are not distinguishable at the sequence level from those of the human host, they are unlikely to be recognized as dietary constituents at all. Actin, for example, is an abundant globular protein that forms microfilaments in muscles (and, therefore, meat), but its sequence is highly conserved among species (e.g. 0 amino acid differences between humans, cows and sheep), and, therefore, it is not possible to assign as a dietary protein. In contrast, the milk protein β-lactoglobulin, which is not encoded by the human genome, is characterized by numerous species-specific amino acid sequence polymorphisms across taxa, for example, 10 differences between cows and sheep.

Furthermore, conclusively demonstrating that animal proteins are derived from ancient dietary sources can be challenging. Laboratory reagents commonly contain a range of enzymes derived from non-human animal proteins and, therefore, laboratory contaminants can easily be misidentified as putative dietary evidence. Here, we took a conservative approach to ruling out common laboratory contaminants from our dataset (discussed below), and as a blanket rule, excluded all collagens, keratins and egg proteins from our list of identified proteins. It is possible that proteins derived from animal sources are present but remain unidentified using our current bioinformatic approaches. Ancient DNA analyses have revealed a range of putative animal DNA sequences within dental calculus [2,26], and, therefore, ancient metagenomics, in combination with metaproteomics, may provide greater insights into the range of animal species consumed.

### (d) Plant proteins

We identified putative plant proteins originating from oats (*A. sativa*), peas (*P. sativum*) and Brassicaceae in archaeological dental calculus and from potato (*S. tuberosum*), soybean (*Glycine*) and peanut (*A. hypogaea*) in modern individuals. We also detected six ancient and 14 modern conserved plant proteins that could only be resolved to broad taxonomic levels, such as rosids and fabids. Plant proteins were identified in samples across the entire dataset, however, those detected in earlier time periods failed to meet our ‘two-peptide' identification criterion. Only within the GASP-extracted post-medieval and modern samples were we able to robustly identify plant-derived food proteins, suggesting that different extractions methods may influence the recovery of plant proteins within dental calculus.

The generally low abundance of plant proteins within the dataset, especially cereal proteins, is intriguing considering the known importance of bread wheat in post-medieval diets in the UK [35]. It is possible that in addition to protein extraction techniques, cooking processes may also influence both the preservation and recovery of plant proteins, for example, through greater rates of Maillard reactions due to the relatively higher proportion of (reactive) sugars in plant tissues [36–38]. Nevertheless, the recovery of plant protein sequences demonstrates that this approach has potential for future investigations of ancient plant consumption. In particular, ancient protein sequence analysis offers a method by which to identify not only plant taxa but also the utilization of specific plant tissues. For example, in the dental calculus of one post-medieval individual from Yorkshire (FW450), we identified peptide sequences specific to the 12S seed storage protein of *A. sativa* (oats), a protein which specifically expressed in the grain of this cereal.

While we know that individuals both today and in the past target the edible seeds of cereals, the fact that shotgun proteomics can identify specific plant tissues is a novel development for understanding plant utilization in past populations. For example, while microscopic analyses of starches within dental calculus have been instrumental in documenting cereal preparation and consumption across multiple geographical areas and time periods [11,39–41], proteomic analyses may uniquely provide insight into those plants that do not produce diagnostic starches, or where the leafy green portion of the plant is consumed. Reference sequence databases upon which this approach depends, however, are highly biased towards commercially significant species, such as key domesticates. Even when ‘dietary' taxa are represented in protein reference databases, they may be constrained to particular classes of proteins or biological pathways. Among entries derived from plant species in the UniProt database, there is a bias towards photosynthetic genes and proteins, or allergenic proteins, which may not represent the full range of plant portions that may have been consumed. For example, in one individual (STB21) we found peptide evidence of an allergenic epitope (Bra j 1-E) expressed specifically within brassica seeds. Although multiple portions of brassica plants are edible, including bulbs, stems, leaves and seeds, restrictions in current reference databases may currently mask our ability to detect the consumption of these other tissues. As databases expand to include a wider variety of plant species and tissues, however, our ability to detect plant consumption may likewise improve.

Analysis of microfossil remains in ancient dental calculus can also reveal evidence of inhaled or ingested plants remains that result from non-dietary sources [8,9], including inhaled pollens, wood particles, charcoal, textile fibres, etc. In this study, we found no evidence of plant proteins which could be assigned to non-dietary sources, nor evidence of plant tissues or species used for textile manufacture (e.g. flax, cotton). This lack of non-dietary plant proteins may be influenced by multiple factors, including: protein diagenesis in particular plant tissues, a lack of analytical sensitivity to detect non-dietary plant proteins, low abundance of proteins in (cellulose and lignin rich) structural tissues, and limitations in current protein databases which focus primarily on proteins of other scientific interests.

### (e) Host digestive enzymes: alpha-amylase

In addition to proteins derived from dietary sources, we also identified the digestion-related protein α-amylase. This salivary digestive enzyme is the first stage in the breakdown of dietary starch through the hydrolysis of 1,4-α-glucoside bonds in oligo- and polysaccharides. Salivary amylase gene (AMY1) copy number, and in turn, amylase protein expression levels, vary significantly between individuals but on average correlate with histories of starch consumption in human populations [42,43]. We detected α-amylase peptides in 48 individuals, representing all time periods except for the Anglo-Saxon period (electronic supplementary material, figure S2). The average number of α-amylase peptides per individual increases through time, suggesting that overall protein preservation may play a role in the detection of this dietary enzyme. Even when the average number of α-amylase peptides are normalized by the total number of identified peptides for each individual, the modern individuals display an increased proportion of amylase peptides compared to previous time periods (electronic supplementary material, figure S2). Although no significant differences were observed in the detection of amylase peptides between time periods (one-way ANOVA, 4,114 = 1.21366, *p* = 0.31), these findings do suggest that quantitative analysis of salivary amylase in dental calculus may be an additional method by which dietary starch consumption can be studied in the future. Nevertheless, further research is necessary to identify the extent to which taphonomic (e.g. protein degradation), biological (e.g. AMY1 copy number variation, amylase enzyme production) and dietary factors (e.g. starch consumption) influence its detection in dental calculus.

## 4. Challenges and recommendations for dietary protein studies

There are several inherent challenges in confidently inferring protein identities in a shotgun proteomic dataset, including the presence of multiple distinct proteins with a high degree of sequence homology [44], and the large number of low-scoring spectral matches [45]. Below, we discuss protein extraction methods, data analysis and authentication strategies that are of particular importance in the identification of dietary proteins from archaeological dental calculus.

### (a) Data generation

#### (i) Extraction methodologies

In this study, we searched for dietary proteins within archaeological dental calculus data obtained from newly generated datasets as well as from a previous study on ancient dairying [3]. For the newly generated datasets, we adopted a new protein extraction method, GASP, based on Fischer & Kessler [27], using dental calculus samples from the post-medieval period and modern individuals. In two of the modern individuals (samples 1004, 1010) where we can compare the efficacy of the two extraction methods on the same starting material, the total protein identifications achieved using the GASP extraction method (*n* = 1159 and 1324 protein hits, respectively) exceeds those achieved using FASP extraction method by an order of magnitude (*n* = 159 and 112 protein hits, respectively). Moreover, a comparison of proteins identified by both methods in these two individuals indicates that the GASP method recovers a much greater diversity of proteins, including virtually all those identified through FASP (electronic supplementary material, figure S3). Although only two paired samples were directly compared, these results demonstrate how extraction method can substantially impact protein recovery, representation and identification.

#### (ii) Monitoring for contamination

The detection of putative dietary-derived proteins in ancient dental calculus should be approached with caution due to the potential for laboratory contamination [46]. There are two factors which may render this analytical approach particularly susceptible to contamination from non-endogenous proteins: (1) the very low proportion of dietary proteins in the biological source; and (2) the presence of modern milk, blood and other proteins in laboratory reagents and standards. For example, caseins are routinely used in western blot analysis, bovine serum albumin is commonly used as a quantitative standard and as a reagent in immunological assays, and egg-derived proteins such as lysozyme and ovalbumin are often included in cell lysis buffers or used as molecular weight markers. Care should be taken to select reagents that are chemically pure and free from proteinaceous components. In addition, laboratory controls including blank extractions and LC–MS/MS injection blanks should be performed in order to monitor for and detect such contamination in laboratory reagents and consumables [17].

### (b) Maximizing dietary identifications while avoiding spurious matches

#### (i) MS/MS search strategies

Within complex samples such as dental calculus, identifying a greater number of peptides, including non-tryptic peptides, can increase protein coverage and improve protein identifications overall [47]. However, search strategies that attempt to maximize protein identifications by including multiple post-translational modifications (PTMs) or enzyme modifications rapidly become unsustainable due to the exponential increase in search space and its impact on the total quantity of information and computational time [48]. Searching for all potential non-tryptic peptides is computationally intensive, as the number of candidate peptide sequences within the database increases dramatically. Additionally, non-tryptic or semi-tryptic searches may ultimately decrease the number of identifications due to a higher false discovery rate resulting from an enlarged search space.

Our initial Mascot search strategy only considered peptides which conformed to tryptic cleavage patterns. To assess the extent to which enzyme modifications influence the identification of putative dietary proteins, we performed a comparison of spectral data searching using tryptic versus semi-tryptic modifications on 36 samples dating from the Iron Age to the medieval period, also using Mascot (electronic supplementary material, table S5). While tryptic searches produced slightly more high-confidence dietary protein identifications (11 versus 9 proteins, all of which were assigned to β-lactoglobulin), semi-tryptic searches identified more non-milk proteins, including one cyprinid and one cereal protein. These results suggest that a greater total number of proteins may be achieved using a combined approach, or by adopting optimized bioinformatic methods for non-tryptic peptide identification [47,49].

#### (ii) Choosing appropriate databases

The identification of dietary proteins from dental calculus is highly influenced by the selection and composition of reference protein sequence databases. Reference databases are composed of protein and translated genomic sequences mostly derived from domesticated, economically relevant species, and these databases contain only a small fraction of the species that exist in nature. The use of an incomplete database may result in false positive matches of conserved sequences to homologous proteins, leading to taxonomic misassignment [46]. The creation of a customized database that includes all potential microbial and eukaryotic species available, however, significantly increases the computational resources required for the project.

UniProt is a commonly used database of non-redundant protein sequences that span all domains of life. Microbial entries within the database, however, are smaller in number than those that can be predicted from current microbial genomic databases. Because the dental calculus proteome is dominated by microbial proteins, it is important to determine whether or not it is necessary to supplement the UniProt database with additional microbial sequences when analysing this substrate. In order to test the impact of database selection on downstream dietary identifications, we searched spectral data generated from a subset of the 19 oldest samples in our study against two different databases: the UniProt database, and a combined database of UniProt and translated protein sequences from the HOMD [2,3,50]. Electronic supplementary material, table S6 displays the proteins and organisms identified by at least one peptide matching to Eukaryotes at the family level or below (excluding *Homo sapiens*). Searching against the UniProt database alone yielded a greater number of protein identifications which could be attributed to dietary sources (*n* = 25), but nearly 80% of these proteins were represented by either a single peptide or multiple peptides representing the same region of the protein. Spectral searching also produced matches specific to model organisms (e.g. *Xenopus laevis* and *Danio rerio*) and other species which were unlikely to be ingested by past individuals (e.g. *Felinae* and the pit viper *Bothrops jararaca*), demonstrating that a high number of false positive taxonomic matches may result when searching against an incomplete database. In contrast, searching against the UniProt database in combination with HOMD, produced fewer dietary protein identifications (*n* = 10), even when including proteins with only a single peptide; however these were dominated by the milk protein β-lactoglobulin, and with no matches to model organisms or unexpected species. These results also demonstrate the importance of having two or more confidently identified peptides for each protein (the so-called ‘two-peptide rule’ [51,52]) to reduce misidentifications and false positive results.

## 5. Conclusion

This study demonstrates that proteomic analysis of ancient dental calculus is a viable approach for recovering dietary information from the archaeological record; nevertheless, there are limitations that have yet to be overcome to maximize the detection of these low-abundance proteins. Through the analysis of 100 archaeological samples spanning the Iron Age to post-medieval England, we detected proteomic evidence of dairy products, cereal grains, legumes and vegetative crops, and demonstrate the value in revisiting proteomic datasets with new methods and approaches [53]. For metaproteomic analyses of complex biological substrates like dental calculus, identification of low-abundance dietary proteins appears to remain fundamentally a stochastic event, influenced by a range of factors including: the initial entrapment of the protein in plaque during life; protein extraction method; protein structure and PTMs; and the dynamic range of the mass spectrometer [45]. The detection of dietary proteins (like other methods of detecting dietary evidence from dental calculus) appears to be serendipitous, and at best, a method for confirming the consumption of particular foods at the population level, rather than investigating particular dietary differences between individuals. Nevertheless, the development of methods to ‘enrich' dietary proteins of interest, using immuno-assays, affinity columns or targeted LC-MS/MS approaches may ultimately increase both the sensitivity and the range of dietary proteins detected in calculus. Finally, this study illustrates how dietary proteins can elucidate foodstuffs that are otherwise invisible by microscopic approaches, such as milk or meat, and enhance the detection of understudied vegetative crops, especially in regions where micro- and macrobotanical remains are poorly studied or not preserved.

## Supplementary Material

Supplementary Information

## Supplementary Material

Supplementary Table 1

## Supplementary Material

Supplementary Table 2

## Supplementary Material

Supplementary Table 3

## Data accessibility

Mass spectrometry data are available via the ProteomeXchange Consortium under the accession PXD009603 [54].
