# Supplementary material for: Proteomic evidence of dietary sources in ancient dental calculus
Source: Proc Biol Sci. 2018 Jul 18;285(1883):20180977. doi: 10.1098/rspb.2018.0977 (PMC6083251; doi:10.1098/rspb.2018.0977)
Supplement: Supplementary Information [file rspb20180977supp1.docx]

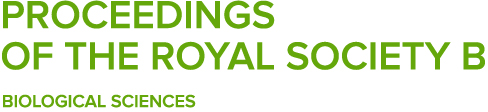


Supplementary Information for

Proteomic Evidence of Diet in Ancient Dental Calculus

Jessica Hendy*, Christina Warinner, Abigail Bouwman, Matthew Collins, Sarah Fiddyment, Roman Fischer, Richard Hagan, Courtney A. Hofman, Malin Holst, Eros Chaves, Lauren Klaus, Greger Larson, Meaghan Mackie, Krista McGrath, Amy Mundorff, Anita Radini, Huiyun Rao, Christian Trachsel, Irina M. Velsko, Camilla F. Speller*.

*Correspondence to: Jessica Hendy (hendy@shh.mpg.de), Camilla Speller (camilla.speller@york.ac.uk)

This pdf includes:

1. Detailed description of methods
2. SI Table 4. Contaminant list
3. SI Table 5. Total protein identifications, as well as putative dietary protein and peptide identifications using semi-tryptic and fully-tryptic peptide identification search strategies.
4. SI Table 6. Putative dietary proteins and peptides identified searching against Uniprot and HOMD databases and searching against the Uniprot database alone.
5. SI Figure 1. Relationship between quantity of dental calculus used for protein extraction and the number of identified proteins.
6. SI Figure 2. Bar chart demonstrating the mean frequency of ‘alpha-amylase 1’ peptides in dental calculus from different archaeological time periods and modern samples.
7. SI Figure 3. Comparison of FASP and GASP extracts from two samples of modern dental calculus.
8. **Detailed Description of Methods**

**Summary.** A total of 100 archaeological samples of dental calculus were analyzed in this study, with an additional 14 deriving from dental calculus deposits of modern or recently deceased individuals. Of these 14 modern individuals, 10 were dental patients and 4 recently deceased individuals from the University of Tennessee Forensic Anthropology Centre. Two extraction methods were performed; a Filter-Aided Sample Preparation Protocol (FASP) previously published in Warinner et al. [[1,2]](https://paperpile.com/c/LiNijR/DNUE0+ZVuQZ/?noauthor=1,1), and a Gel-Aided Sample Preparation Protocol (GASP) based on Fischer and Kessler [[3]](https://paperpile.com/c/LiNijR/283PW/?noauthor=1) modified for ancient mineralized samples. This latter extraction method was applied in order to improve the number of identified proteins from ancient and modern samples. Protein extractions were performed at three locations; the ancient protein laboratory at BioArCh, at the Department of Archaeology, University of York (UK), laboratories at the Department of Archaeology, University of Oxford, and at the Laboratories of Molecular Anthropology and Microbiome Research at Oklahoma University (USA). MS/MS analysis was performed in two locations; the mass spectrometry laboratories at the Target Discovery Institute (University of Oxford, United Kingdom), and at the Functional Genomics Center Zürich (ETH Zürich/University of Zürich, Switzerland). The extraction procedure, location of extraction and location of MS/MS analysis for each sample is summarized in Supplementary Table 1.

**Re-Analysis of Published Datasets.** We analysed 38 existing datasets previously published in Warinner et al. [[1]](https://paperpile.com/c/LiNijR/DNUE0/?noauthor=1) focussing on populations from the UK. Raw data were downloaded from ProteomeXchange (Accession ID: PXD001362) and converted to mgf files using Proteowizard MSConvert (version 3.0.4743) using the 200 most intense peaks in each MS/MS spectrum. MS/MS ion database searching was performed on Mascot (Matrix Science^TM^, version 2.4.01), against UniProt and the Human Oral Microbiome database previously published in Warinner et al. [[1]](https://paperpile.com/c/LiNijR/DNUE0/?noauthor=1). Searches were filtered to display peptides with an ion score of greater than 25, and estimated protein false discovery rates were adjusted to 5%.

**Protein Extraction: Gel Aided Sample Preparation Protocol.** We selected 62 samples of dental calculus from post-medieval contexts in England and extracted proteins using a Gel-Aided Sample Preparation protocol based on Fischer and Kessler [[3]](https://paperpile.com/c/LiNijR/283PW/?noauthor=1) modified for ancient mineralized samples. Sample were ground to a powder using sterile micropestles, then decalcified for 5 minutes with 1 mL of 0.5M EDTA to remove possible surface contamination. This pre-digestion supernatant was removed, and a further 1 mL of 0.5M EDTA added, and rotated at room temperature for seven days to fully demineralize. Samples were centrifuged at 13,000 RPM for 2 minutes and 950 μL of supernatant transferred into a new eppendorf tube, while the pellet and remaining supernatant was retained for proteomic analysis. For samples from Fewston (Sample code: FW) the total 1 mL of EDTA decalcified solution was used for proteomic analysis.

To the pellet and remaining 50 μL of EDTA, 5 μL of SDS (20%) was added with 45 μL of B-PER (Bacterial Protein Extraction Reagent, Thermo Fisher), then shaken for 15 minutes at room temperature. 50 μL of DTT (1M) was added and the samples shaken for a further 30 minutes at room temperature. 100 μL of Proto-Gel (30%, National Diagnostics) was added and gently resuspended to mix, then left on the benchtop for 20 minutes. To polymerize the gel, 8 μL of tetramethylethylenediamine (TEMED), followed by 8 μL of 10% ammonium persulfate (APS) was added and gently mixed. The polymerized gel was then shredded into pieces to increase the surface area by passing the gel through a plastic grid inset by pulse centrifugation. The gel pieces were fixed through the addition of methanol/water/acetic acid/ solution (50/40/10). The solution was centrifuged, and the supernatant discarded. 1 mL of acetonitrile was added to dehydrate the gel pieces. A series of washing and drying steps using acetonitrile were then performed to exchange buffers. 1 mL of urea (6M) was added to the dehydrated gel pieces and rotated for 3 minutes. 1 mL of acetonitrile was added to partially dehydrate, rotated for 3 min, briefly centrifuged, then the supernatant removed. A further 1 mL of acetonitrile was added to fully dehydrate the gel pieces. A further 1 mL of urea (6M) was added and subsequently removed using acetonitrile dehydration. To the dried gel pieces, 1 mL of ammonium bicarbonate (0.05M) was added and rotated for 3 minutes. 1 mL of acetonitrile was added to partially dehydrate, rotated for 3 minutes, briefly centrifuged, then the supernatant removed. A further 1 mL of acetonitrile was added to fully dehydrate the gel pieces. 200 μL of ammonium bicarbonate (0.05M) and trypsin (5 μL of 0.5 μg/μL) were added and left to digest at 37 °C overnight.

The following morning, digested peptides were extracted from the gel. 200 μL of acetonitrile was added to dehydrate the gel pieces, samples were rotated for 5 minutes, pulse centrifuged, then the supernatant transferred to a new tube. 200 μL of 5% formic acid solution was added to the gel pieces to extract acidic peptides from the gel pieces, rotated for 5 minutes, then pulse centrifuged. 200 μL of acetonitrile was added to the gel pieces, rotated for 5 minutes, pulse centrifuged, and the supernatant transferred to the tube containing the first fraction. To fully dehydrate the gel, a further 200 μL of acetonitrile was added, rotated for 5 minutes, pulse centrifuged, and the supernatant transferred to the sample tube. After drying in a centrifugal evaporator all samples of extracted peptides, apart from FW samples and Z100 and Z90 were desalted using Millipore Zip-Tips prior to MS/MS analysis.

**Modern Samples.** We also extracted proteins from samples of modern dental calculus including US dental patients (n=10) and individuals from recent human skeletal material from the Forensic Anthropology Centre, University of Tennessee (n=4). Samples from the University of Tennessee were extracted following a the FASP method described in Warinner et al. [[1]](https://paperpile.com/c/LiNijR/DNUE0/?noauthor=1), but with separate extracts from supernatant and pellet fractions. Samples from dental patients were extracted using FASP previously published in Warinner et al. [1], although two samples (OU1005, OU1010) were subsampled, with one fraction extracted using FASP and the other with GASP, enabling a paired comparison of GASP and FASP extractions for modern samples.

**nLC-MS/MS Analysis.** Tryptic peptides were analysed at two locations: at the Mass Spectrometry Laboratories of the Target Discovery Institute at the University of Oxford using a n-LC Q-Exactive tandem mass spectrometer according to previously published specifications [[1]](https://paperpile.com/c/LiNijR/DNUE0) and at the Functional Genomics Center Zurich of the University/ETH Zurich using the following instrument setup. Samples were analyzed on a Q-Exactive HF mass spectrometer (Thermo Scientific, Bremen, Germany) coupled to an ACQUITY UPLC M-Class system (Waters AG, Baden-Dättwil, Switzerland). Full scan MS spectra were acquired from 375-1500 m/z with an automatic gain control target of 3e6, an Orbitrap resolution of 60,000 (at 200 m/z), and a maximum injection time of 15 ms. Precursor for ms2 scans where isolated with the quadrupole with a window of 1.2 Da, an automated gain control value of 1e5, a maximum fill time of 50 ms and HCD fragmented with a normalized collision energy of 28. From each MS1 scan the 12 most intense precursor ions were fragmented and scanned with a resolution of 30’000 (at 200 m/z) and a fixed first mass of 130 m/z. Filter criteria for MS2 selection were an intensity threshold of 9e4 and unassigned and singly charged ions were excluded. Selected precursor ions were put onto a dynamic exclusion list for 20 seconds. Peptide separation was achieved by RP-HPLC on C18 column (in house packed, 150 mm x 75 µm, 1.9 µm, Reprosil pure C-18 AQ, 120 Ǻ (Dr. Maisch GmbH)). Solvent composition at the two channels was 0.1% formic acid in H2O for channel A and 0.1% formic acid in acetonitrile for channel B. Column temperature was 50°C. 4 µL for each samples were loaded onto the column for 10 minutes with 100% solvent A at 1 µL/min. Composition and flow was changed to 98 % solvent A and 0.3 µL/min and allowed to stabilize for 3 minutes afterwards. Peptides were separated over 60 minutes with a linear gradient of 2 – 40 % B. Column was cleaned after the run by increasing to 98% B in 2 minutes and holding 98 % B for 15 minutes prior to re-establishing loading condition.

**Data Analysis.** Raw spectral data was converted to Mascot generic format (mgf) using Proteowizard MSConvert (version 3.0.4743) using the 200 most intense peaks in each MS/MS spectrum. MS/MS ion database searching was performed on Mascot (Matrix Science^TM^, version 2.4.01), against UniProt (release 2017_06) and the Human Oral Microbiome database previously published in Warinner et al. [[1]](https://paperpile.com/c/LiNijR/DNUE0/?noauthor=1). Searches were performed against a decoy database to estimate protein false discovery rates, which were adjusted to less than 5%. For samples extracted using GASP, propionamide (C) was set as a fixed modification and acetylation (protein N-terminus), deamidation (NQ), methionine oxidation, propionamide (K) and propionamide (N-terminus) were set as variable modifications. For samples extracted using FASP post-translational modifications were set as carbamidomethylation (fixed modification), and acetylation (protein N-term), deamidation (NQ), glutamine to pyroglutamate, and methionine oxidation as variable modifications. Peptide tolerance was 10 ppm, and MS/MS ion tolerance was 0.07 Da. Tryptic peptides were searched with up to 1 missed cleavage; semi-tryptic peptides were also searched with up to 1 missed cleavage for a subset of the samples. Protein results were filtered to a false discovery rate of less than 5% and an ion score of >25, and containing a minimum of two distinct peptides matching to different regions of the protein. To characterise differences in dietary protein identifications using different search databases, MS/MS ion database searching was also performed using Mascot (Matrix Science^TM^, version 2.4.01), against the UniProt database only, using the same parameters listed above.

Based on initial Mascot results, we used an R script to assign proteins by taxon/protein name into the following classifications: Contaminant, Non-Human Animal, Human, and Plant (main text, Table 2). We took a conservative approach and assigned any protein identified in our blank controls or injection blanks to the ‘contaminant’ category. Initially, all non-human animal and plant peptides were considered as deriving from potential dietary proteins, and were further interrogated using BLAST (NCBI). Peptides were aligned using BLASTp against all non-redundant nucleotide sequences. Any non-human animal or plant peptides that also matched identically to human or microbial proteins were eliminated as possible dietary proteins. Likewise, any non-human animal or plant peptides deriving from proteins identified within the ‘contaminant’ dataset (SI Table 4) were also eliminated as as potential dietary proteins. Protein expression data (tissue or cell specificity) was retrieved from UniProtKB where available. Statistical analyses were conducted using SPSS (v. 23, IBM Corporation). We assessed normality of the data using the Shapiro-Wilks test and tested assumption of homogeneity of variances using Levene's test of equality of variances. When comparing total protein identifications and alpha-amylase 1 peptides through time, we applied Welch's ANOVA, followed by Games-Howell post hoc tests.

**Contamination controls and data authentication.** Several precautionary measures were taken to reduce contamination and ensure that the reported proteins were endogenous to the dental calculus [[4]](https://paperpile.com/c/LiNijR/uHH3), including: 1) the use of dedicated ancient protein laboratories where modern proteins are not extracted or analysed; 2) the inclusion of blank controls to monitor for contamination during protein extraction; 3) the inclusion of injection blanks between each dental calculus sample to monitor for protein carryover during LC-MS/MS; and 4) stringent MS/MS ion search criteria and inclusion criteria (e.g., two unique peptides, verification through BLASTp searches).

1. **Contaminant List**

**SI Table 4.** A list of contaminants was compiled based on: a) previous reports of contaminating proteins in previous studies [[1]](https://paperpile.com/c/LiNijR/DNUE0); and b) detection of these proteins in extraction and instrument blanks.

| **Protein** | **Species** | **Reason** |
| --- | --- | --- |
| Lysozyme, Ovalbumin | From any species | Laboratory Reagent and Common Contaminant |
| Keratins | From any species | Skin |
| Collagens | From any species | Skin |
| Albumin | *Bos taurus* | Laboratory Reagent |
| Green fluorescent protein | *Aequorea victoria* | Laboratory Reagent |
| Rubber elongation factor protein | *Hevea brasiliensis* | Latex Gloves |
| Dermcidin | *Homo sapiens* | Skin |
| Tubulin | From any species | Common Contaminant |
| Actin | From any species | Common Contaminant |
| Any Saccharomyces protein | *Saccharomyces* | Dust/Environment |
| S100-A8 | From any species | Detection in extraction blank |
| Glutathione S-transferase class-mu 26 kDa isozyme | *Schistosoma* | Detection in extraction blank |
| Bifunctional protein GlmU | *Acidiphilium* | Detection in extraction blank |
| Alpha-1-acid glycoprotein | From any species | Detection in extraction blank |
| Desmoplakin | From any species | Skin |
| Alpha-2-HS-glycoprotein (Fetuin) | From any species | Laboratory Reagent |
| Caspase-14 | From any species | Skin |

1. **Comparison of dietary proteins identified using tryptic and semi-tryptic searches**

**SI Table 5.** Comparison of total protein identifications, as well as putative dietary protein and peptide identifications searching semi-tryptic and tryptic peptides from the same sample.

| **Sample** | **Period** | **Semi Tryptic** | | **Tryptic** | |
| --- | --- | --- | --- | --- | --- |
|  |  | **Total IDs** | **Dietary Protein (number of peptides)** | **Total IDs** | **Dietary Protein (number of peptides)** |
| ML1489 (CS1) | Iron Age | 44 |  | 62 |  |
| ML1823 (CS2) | Iron Age | 106 |  | 158 | Beta-lactoglobulin, Bovidae (17) |
| ML3890 (CS3) | Iron Age | 20 |  | 52 |  |
| ML1032 (CS4) | Iron Age | 86 | Beta-lactoglobulin, Bovidae (8); Pyruvate, phosphate dikinase, Oryza sativa (3) | 109 | Beta-lactoglobulin, Bovidae (8) |
|  |  |  |  |  |  |
| OX01 | Roman | 58 |  | 96 | Beta-lactoglobulin, Bovinae (2) |
| OX03 | Roman | 64 |  | 79 |  |
| OX04 | Roman | 87 | Beta-lactoglobulin, Bovidae (12) | 106 | Beta-lactoglobulin, Bovidae (7) |
| OX05 | Roman | 42 |  | 37 |  |
| OX06 | Roman | 90 |  | 100 |  |
| OX09 | Roman | 33 |  | 38 |  |
| OX10 | Roman | 126 |  | 126 |  |
| OX12 | Roman | 116 | Beta-lactoglobulin, Ovi-caprid (22) | 169 | Beta-lactoglobulin, Bovidae (10) |
| 3DT26 (CS5) | Roman | 55 |  | 165 |  |
| 3DT21 (CS6) | Roman | 100 | Beta-lactoglobulin, Bovinae (3) | 110 | Beta-lactoglobulin, Bovidae (16) |
| 6DT3 (CS7) | Roman | 107 |  | 186 |  |
| 6DT7(CS8) | Roman | 36 |  | 52 |  |
| 6DT21 (CS9) | Roman | 18 |  | 20 |  |
| HE1987 | Roman | 24 |  | 31 |  |
| HE2000 | Roman | 46 | Collectin-12, Cyprinid (3) | 13 |  |
| JV15548 (CS12) | Viking | 68 |  | 132 | Beta-lactoglobulin, Bovidae (4) |
| NEM18 (CS13) | Anglo-saxon | 68 | Beta-lactoglobulin, Bovidae (20) | 81 | Beta-lactoglobulin, Bovidae (20) |
| NEM093 (CS14) | Anglo-saxon | 23 |  | 52 |  |
| NEM099 (CS15) | Anglo-saxon | 88 |  | 160 |  |
| NBS410 (CS16) | Anglo-saxon | 33 |  | 48 |  |
| NBS262 (CS17) | Anglo-saxon | 87 |  | 163 |  |
| NBS325 (CS18) | Anglo-saxon | 27 |  | 39 |  |
| TKAC (CS19) | Medieval | 64 |  | 115 |  |
| TKDC (CS20) | Medieval | 117 |  | 146 |  |
| TKEC (CS21) | Medieval | 97 |  | 145 |  |
| TKFC (CS22) | Medieval | 70 |  | 82 |  |
| WG1252 | Medieval | 222 |  | 272 |  |
| WG1082 | Medieval | 251 | Beta-lactoglobulin, Bovidae (7) | 331 | Beta-lactoglobulin, Bovidae (5) |
| WG1566 | Medieval | 189 |  | 221 |  |
| WG1483 | Medieval | 155 |  | 202 |  |
| WG1561 | Medieval | 178 | Beta-lactoglobulin, Bovidae (4) | 221 | Beta-lactoglobulin, Bovidae (5) |
| WG1585 | Medieval | 130 |  | 184 | Beta-lactoglobulin, Bovidae (2) |

1. **Comparison of dietary proteins identified using UniProt and UniProt + HOMD**

**SI Table 6.** Comparison of putative dietary proteins and peptides identified searching against Uniprot and HOMD databases and searching against the Uniprot database alone. Shaded cells indicate proteins identified on the basis of only one peptide sequence.

| **Sample** | **Period** | **Uniprot**  **Protein (# of peptides)** | **Uniprot & HOMD**  **Protein (# of peptides)** |
| --- | --- | --- | --- |
| ML1489 (CS1) | Iron Age | Beta-lactoglobulin, Bovidae (5) | Beta-lactoglobulin, Bovidae (5) |
| ML1823 (CS2) | Iron Age | Beta-lactoglobulin, Bovidae (17) | Beta-lactoglobulin, Bovidae (17) |
|  | Iron Age | Protein phosphatase 1 regulatory subunit 32, Bovidae (3) |  |
| ML3890 (CS3) | Iron Age |  |  |
| ML1032 (CS4) | Iron Age | Beta-lactoglobulin, Bovidae (8) | Beta-lactoglobulin, Bovidae (8) |
| OX01 | Roman Period | Beta-lactoglobulin, Bovinae (2) | Beta-lactoglobulin, Bovinae (2) |
| OX03 | Roman Period | CASP-like protein 2C2, Oryza sativa (1); RNA pseudouridylate synthase domain-containing protein, Danio rerio (1) |  |
| OX04 | Roman Period | Beta-lactoglobulin, Bovidae (7) | Beta-lactoglobulin, Bovidae (7) |
| OX05 | Roman Period |  |  |
| OX06 | Roman Period | Beta-lactoglobulin, Bovinae (2); Bloom syndrome protein homolog, Galloanserae (2) | Beta-lactoglobulin, Bovinae (2) |
| OX09 | Roman Period |  |  |
| OX10 | Roman Period | Chromodomain-helicase-DNA-binding protein 8, Cyprinidae (1); Vacuolar protein sorting-associated protein 26B-B, Xenopus laevis (1); F-box/kelch-repeat protein At4g19870, Camelinaea (6); Putative glycerol-3-phosphate transporter 2, Arabidopsis thaliana (4); 3-oxoacyl-[acyl-carrier-protein] synthase III, chloroplastic, Spinacia oleracea (2) |  |
| OX12 | Roman Period | Beta-lactoglobulin, Bovidae (10) | Beta-lactoglobulin, Bovidae (10) |
|  |  | UDP-glycosyltransferase 88B1, Panicoideae (1) | UDP-glycosyltransferase 88B1, Panicoideae (1) |
| 3DT26 (CS5) | Roman Period |  |  |
| 3DT21 (CS6) | Roman Period | Beta-lactoglobulin, Bovidae (16) | Beta-lactoglobulin, Bovidae (16) |
|  |  | 1-aminocyclopropane-1-carboxylate oxidase 4, Camelineae (7) |  |
| 6DT3 (CS7) | Roman Period | Glyceraldehyde-3-phosphate dehydrogenase 2, Agaricus bisporus (3); Protein MRG2, Brassicaceae (3) | Beta-lactoglobulin, Bovinae (4) |
| 6DT7(CS8) | Roman Period | Taxadiene synthase, Taxus (1) |  |
| 6DT21 (CS9) | Roman Period |  |  |
| HE1987 | Roman Period | Retinoid isomerohydrolase, Cyprinidae (1) |  |
| HE2000 | Roman Period | Bradykinin-potentiating peptide 11i, Bothrops jararaca (1); Steroid 21-hydroxylase, Felinae (2) |  |


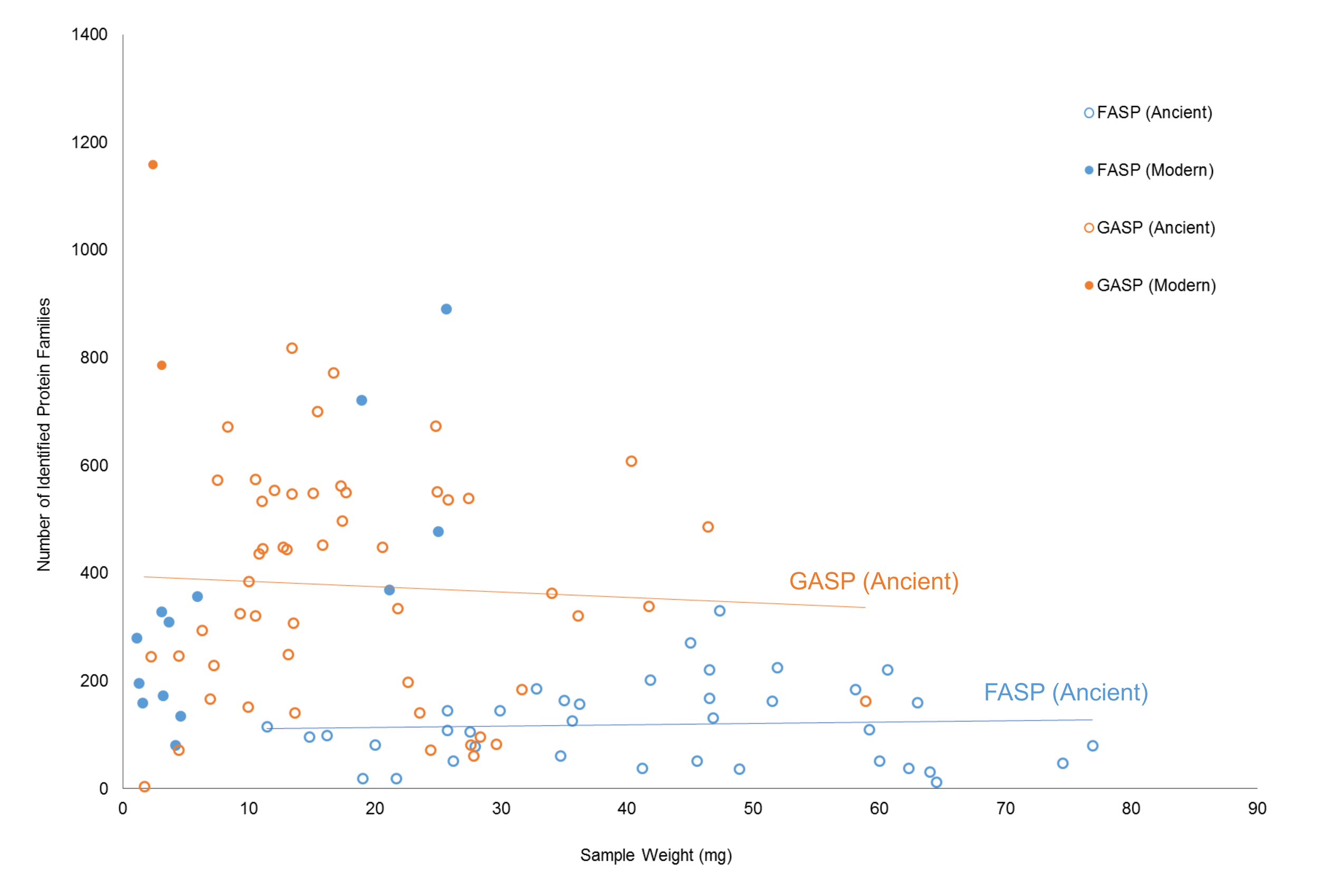


**5. SI Figure 1.** Relationship between quantity(mg) of starting material and number of identified proteins. Pearson's product-moment correlation displayed no significant correlation between starting weight and total number of identified proteins in either ancient samples extracted with either the FASP (r(38)=0.056, p=0.74) or GASP (r(52)=-0.057, p=0.686) method.


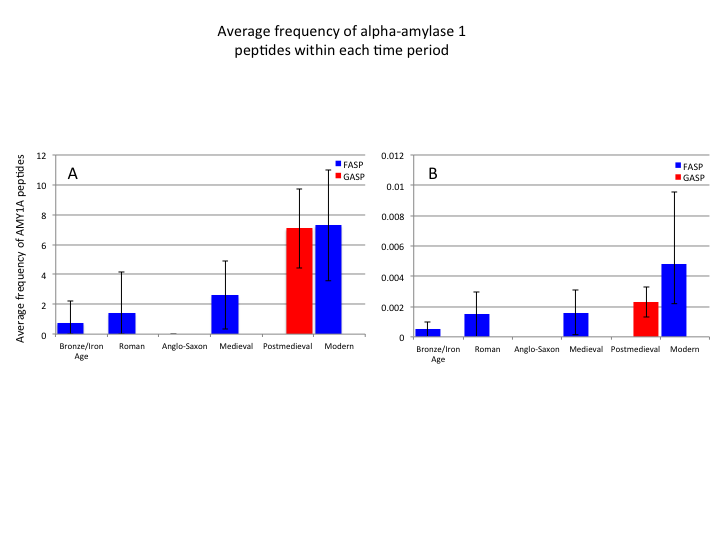


**6. SI Figure 2.** Bar chart displaying the mean frequency of alpha-amylase 1 peptides in dental calculus for archeological and modern individuals (A, raw counts and B, as a proportion of total identified peptides). Error bars indicate 95% confidence intervals.


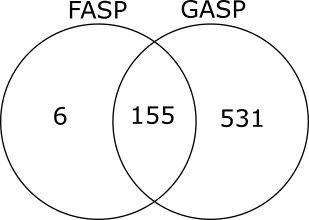


**7. SI Figure 3.** Comparison of FASP and GASP extracts pooled from two modern dental calculus samples (1004, 1010). Data was generated by searched mzIdentML files in Mascot against the UniProt database in Scaffold (Proteome Software Inc.). Protein identifications were generated with a protein threshold of 99.0%, a peptide threshold of 95% and with a minimum of two peptides. FASP and GASP generated files were pooled in two biological categories and counts of individual proteins compared.

**References**

1. [Warinner C *et al.* 2014 Direct evidence of milk consumption from ancient human dental calculus. *Sci. Rep.* **4**, 7104.](http://paperpile.com/b/LiNijR/DNUE0)

2. [Warinner C *et al.* 2014 Pathogens and host immunity in the ancient human oral cavity. *Nat. Genet.* **46**, 336–344.](http://paperpile.com/b/LiNijR/ZVuQZ)

3. [Fischer R, Kessler BM. 2015 Gel-aided sample preparation (GASP)--a simplified method for gel-assisted proteomic sample generation from protein extracts and intact cells. *Proteomics* **15**, 1224–1229.](http://paperpile.com/b/LiNijR/283PW)

4. [Hendy J, Welker F, Demarchi B, Speller C, Warinner C, Collins MJ. 2018 A guide to ancient protein studies. *Nat Ecol Evol* (doi:](http://paperpile.com/b/LiNijR/uHH3)[10.1038/s41559-018-0510-x](http://dx.doi.org/10.1038/s41559-018-0510-x)[)](http://paperpile.com/b/LiNijR/uHH3)
